# Laboratory markers of cardiac and metabolic complications after generalized tonic-clonic seizures

**DOI:** 10.1186/s12883-017-0965-4

**Published:** 2017-09-19

**Authors:** Robert D. Nass, Sina Meiling, René P. Andrié, Christian E. Elger, Rainer Surges

**Affiliations:** 1 Department of Epileptology, University Hospital Bonn, Bonn, Germany; 2 Department of Medicine - Cardiology, University Hospital Bonn, Bonn, Germany; 3 Department of Neurology, Section of Epileptology, RWTH University Hospital Aachen, Aachen, Germany

**Keywords:** Generalized tonic-clonic seizures, Creatine kinase, Injuries, Myocardial infarction, Troponine, Epilepsy

## Abstract

**Background:**

Generalized tonic-clonic seizures (GTCS) frequently lead to emergency inpatient referrals. Laboratory blood values are routinely performed on admission to detect underlying causes and metabolic or cardiac complications. Our goal was to assess the nature and frequency of complications occurring in association with GTCS.

**Methods:**

We retrospectively extracted data from emergency protocols and discharge letters of adult patients admitted to the Department of Epileptology between 01/2010 and 06/2015. Inclusion criteria were diagnosis of GTCS and admission via emergency services. Exclusion criteria were status epilepticus prior to admission to hospital and non-generalized seizures.

**Results:**

A total of 223 patients (of 986 screened cases) were included. Overall, 1.8% required intubation while 1.3% had less severe respiratory problems. In 5.6% of patients, a transient hypoxemia was measured. Hypertensive urgencies affected 7.8% of the patients, sinus tachycardia occurred in 41.2%. Troponin I (cTNI) was determined in 75 patients and was increased in 12% of these cases. Occurrence of elevated cTNI levels was significantly correlated with patient’s age. Four patients were diagnosed with NSTEMI and one patient with STEMI. Creatine kinase (CK) was increased in 59.4% of the patients, with <5-fold increases in 47%, <10-fold in 5.8% and >10-fold increases in 4.3%. Rhabdomyolysis with an >50 fold increase in CK was detected in 1.9% of patients. Prolonged disturbances of consciousness affected 5% of cases while agitation, delirium, and psychotic episodes occurred in 6.3%. Minor traumatic injuries affected 45.7% of patients.

**Conclusions:**

Troponin elevations in association with GTCS are one of the more common complications after emergency admissions especially in older patients. In our selected patient population, serious complications such as intracranial hemorrhage, myocardial infarction and acute renal failure occurred in <1% of GTCS only.

## Background

Seizures are among the most common neurological emergencies and cause about 1% of all emergency room (ER) visits. Most seizures that lead to referral to ERs in adults are acute symptomatic and most in children are febrile convulsions [1]. The most urgent and dramatic type of seizures are generalized tonic-clonic seizures (GTCS). The majority of GTCS are benign but some lead to complications such as cardiac arrhythmias, fractures, rhabdomyolysis, aspiration pneumonia, acute kidney injury and behavioral problems such as delirium or psychosis. Routine laboratory and imaging studies are helpful to identify possible complications and to avoid sequelae. It is, however, widely debated which examinations need to be routinely performed [1–15]. Most studies on seizures in the emergency department have focused on all seizure types, with complication rates and prognosis mostly depending on the underlying pathology of acute symptomatic seizures (e.g. alcohol withdrawal seizures, acute traumatic brain injury and intracranial hemorrhage). The present study aimed to investigate the frequency of laboratory changes and complications after GTCS with an emphasis on people with known chronic epilepsy.

## Methods

### Setting and design

The Department of Epileptology in Bonn is a tertiary epilepsy care center in which adult and pediatric patients with known seizure disorders are treated after elective admission. Emergency patients with known epilepsy are also admitted if they have recurrent seizures, status epilepticus and acute, epilepsy-related health problems. With some variability, about 20% of the patients were admitted via emergency services. In contrast, adult patients with first seizures are routinely treated by the Department of General Neurology and only occasionally by the Department of Epileptology. Children with acute seizures are treated by the Department of Pediatrics. In the present study, only patients who were admitted to the Department of Epileptology via emergency services as well as patients who were transferred to the Department of Epileptology from other hospital’s emergency rooms were included. Exclusion criteria comprised status epilepticus prior to admission to hospital, simple or complex partial seizures, psychogenic, non-epileptic episodes, a chief complaint other than seizures, coding errors with mislabeling of patients as emergencies or an emergency presentation for chronic or subacute problems such as increased seizure frequency, medication side effects etc.

In order to identify emergency admissions, a two-pronged approach was applied. First, the medical controlling department provided a list of all emergency admissions and transfers to our department from January 2010 until June 2015. Second, a full text search of discharge summaries was performed in our electronic patient record database (OS:4.x - client, version 8.0, Optimal Systems GmbH) using the search words “emergency” and “rescue service”. Data were extracted by reading emergency service protocols, discharge summaries and laboratory reports. Only cases in which the initial suspicion of a GTCS was confirmed in the discharge summary were included. In case of uncertainties and doubts about the proper diagnosis the patient was not included in the study. In case of multiple admissions of an individual patient, only the first admission was included in the analysis. Because of its retrospective design, this study was waived from approval by the local ethics committee.

The following demographic features were systematically recorded from patient files: age, sex, epilepsy type or syndrome, comorbidities, cardiovascular risk factors, medications, initial heart rate, blood pressure and oxygen saturation, mental status, intubation, laboratory findings (creatine kinase, cardiac troponin I, creatinine and leukocytes), emergency imaging, injuries. The following outcome features were analyzed: cardiac troponin I (cTNI) elevations, rhabdomyolysis and acute kidney injury as described by the acute kidney injury network criteria (AKIN) [16], in which stage 1 AKI is defined by a 1.5 fold or >0.3 mg/dl increase of creatinine over the course of 48 h, stage 2 by a 2-fold creatinine increase and stage 3 by a 3-fold or >4 mg/dl increase. As non-laboratory parameters, prolonged disturbances of consciousness/vigilance, injuries, respiratory problems and psychiatric complications were assessed.

### Laboratory measurements

cTNI was measured using the cTNI Flex® reagent cartridge, creatine kinase was measured using the CKI Flex® reagent cartridge and creatinine was measured using the CRE2 Flex® reagent cartridge on a Dimension Vista® System, Siemens Healthcare Diagnostics®. The lowest measurement on the cTNI Flex cartridge is 0.02 ng/ml, values above 0.05 ng/ml were considered as elevated. The values of CK were normalized to sex-specific reference ranges.

### Statistics

Normal distribution was tested by Kolmogorov-Smirnov test. To compare relative frequencies of qualitative parameters contingency tables and χ^2^ tests were applied if more than two categories were compared and Fisher’s exact test if two categories were compared. In order to identify differences between groups regarding metric parameters, univariate analysis or non-parametric tests (Mann-Whitney-U- and Kruskall-Wallis-tests) were applied. The Bonferroni-Holmes method was used to correct for errors due to multiple testing. All statistical analyses were performed using IBM® SPSS® Statistics Version 24. 

## Results

### Screening, inclusions and exclusions of patients

**Fig. 1:**
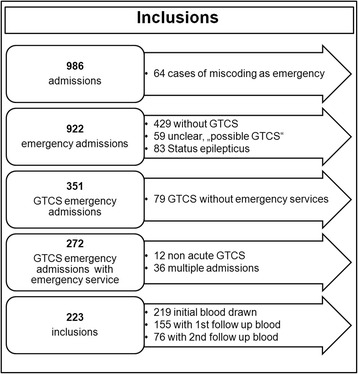
Flow-chart of patient screening, inclusion and exclusion

A total of 986 emergency admissions were identified, of which 64 were no true postictal emergencies but rather urgent admissions due to increasing seizure frequencies, adverse events of anticonvulsant drugs or other health problems. 429 patients had seizure types other than GTCS. In 59 patients, the initial suspicion of GTCS was not confirmed in the discharge summary but changed to other seizure types, psychogenic seizures or syncope. 83 cases had status epilepticus. Figure 1 shows a summary of the screening process.

### Demographics

Our study population consisted of 95 women (42.6%) and 128 men (57.4%). The age ranged from 13 to 75 with a median of 45 years. A history of epilepsy was known in 87.9% with a duration of 7 years (0–64 years). 22 patients (9.9%) had a first seizure and 5 patients (2.2%) were diagnosed as having a substance withdrawal seizure. 27.7% had at least one vascular risk factor (hypertension, diabetes, dyslipidemia, obesity, chronic renal failure) and 26% a cardiovascular disease (coronary artery disease, congestive heart failure, stroke, peripheral vascular disease). 42 (18.8%) patients had a series of GTCS and 181 (81.2%) a single GTCS. Temporal lobe epilepsy (17%) and idiopathic generalized epilepsy (7.2%) were the most common epilepsy forms. However, both the epileptogenic zone and the etiology were unknown in almost 60% of the cases. Patients stayed for a median of 4.9 days (0–35 days) on the ward. 96% returned home thereafter, 4% needed a transfer to psychiatric wards or medical wards, where two patients (0.9%) died due to complications of an underlying medical condition. A summary of patient demographics is provided in Table 1.

**Table 1:** Overview of demographic data

Sex:	Women 95 (42.6%)	Men 128 (57.4%)	
Number of seizures:	181 with single GTCS (81.2%)	42 with seizure series (18.8%)	
Age:	Mean 45 ± 19.2 years	Median 44 years	range 13–75 years
Vascular risk factors:	None: 161 (72.2%)	One: 50 (22.4%)	Multiple: 12 (5.3%)
Seizure history:	1st seizure: 22 (9.9%)	Known Epilepsy: 196 (87.9%)	Substance withdrawal seizure: 5 (2.2%) (4 alcohol, 1 benzodiazepines)
Duration of epilepsy:	Mean 13.1 ± 7 years	Median 7 years	range 0–64 years
No. of current AED:	Mean 1.17 ± 1 AED	Median 1 AED	0–6 AED
Length of stay:	Mean 4.6 ± 3 days	Median 4.9 days	0–35 days
Discharge or transfer to:	Home: 214 (96%)	Dept. Medicine: 5 (2.2%)	Dept. Psychiatry: (1.8%)
Epileptogenic zone:	unclassified: 124 (55.6%)	Temporal: 38 (17%)	Generalized: 23 (10.3%)
	Frontal: 9 (4%)	Occipital 1 (0.4%)	Parietal: 1 (0.4%)
	Multifocal: 19 (8.5%)	Hemispheric: 5 (2.2%)	Other: 2 (0.9%)
Seizure types:	GTCS only: 125 (56.1%)	GTCS and absence: 16 (6.8%)	GTCS and myoclonus: 4 (1.8%)
	GTCS and SPS: 12 (5.3%)	GTCS, SPS and CPS: 64 (28.7%)	LGS (GTCS, TS, CPS): 2 (0.9%)
Etiology:	Unknown: 127 (57%)	Idiopathic/genetic: 16 (7.2%)	Hippocampal Sclerosis: 12 (5.4%)
	Perinatal brain damage: 11 (4.9%)	Posttraumatic: 9 (4%)	Vascular malformation: 9 (4%)
	Postischemic: 7 (3.1%)	Postinfectious: 5 (2.2%)	Other lesions: 4 (1.8%)
	Unspecific lesion: 4 (1.8%)	Cortical malformation: 5 (2.2%)	Neoplastic: 3 (1.3%)
	Posthemorrhagic: 2 (0.9%)	Lennox Gastaut Syndrome: 2 (0.9%)	Immune mediated: 1 (0.4%)
seizure frequency:	GTCS	Persistent (>1 in 6 months): 57 (25.3%) Rare (< 1 in 6 months): 112 (49.8%) Undefined: 54 (24.2%)	
	Other seizure types	Daily: 11 (4.9%) Persistent (>1 in 6 months): 27 (12.1%) Rare (< 1 in 6 months): 130 (58.3%) Undefined: 54 (24.2%)	
Preexisting conditions	All cardiovascular risk factors 62 / 223 (27.8%)	Cardiovascular conditions (CAD, structural heart disease, arrhythmia, history of stroke, peripheral vascular disease): 58 /223 (26%)	

### Vital signs on admission at the ER

The vital signs as documented by the emergency services (information available in 194 patients, for details see Table 2) were assessed. Critical blood pressure values >180 mmHg systolic were found in 15 of 192 patients (7.8%). About 41 % of the patients displayed sinus tachycardia (heart rates exceeding 100 bpm), while critical tachycardia (defined as heart rates exceeding the individual theoretical maximum heart rate according to the formula “220-patient’s age”) only affected 2 of 194 patients (1%). Hypoxia (<90% SpO_2_) was found in 11 of 188 patients (5.8%), critical hypoxia (<71% SpO_2_) in 2 patients (1.1%). Since the documentation of vital signs was incomplete and timing of documentation varied greatly, these features were not submitted to further statistical analysis.

**Table 2:** Summary of vital signs recorded by the emergency services

Feature	Systolic blood pressure	Heart rate	Oxymetric SpO_2_
Availabe n	192 (85.6%)	194 (87%)	188 (84.3%)
Class	Hypotension <100 mmHg: 4 (2.1%)	Bradycardia <60 bpm: 2 (1%)	Normoxia 91–100% SpO_2_: 177 (94.1%)
	Normotension 100–140 mmHg: 110 (57.3%)	Normocardia 60–100 bpm: 108 (55.7%)	Mild hypoxia 86–90% SpO_2_: 6 (3.2%)
	Hypertension 140–180 mmHg: 59 (30.7%)	Tachycardia >100 bpm: 80 (41.2%)	Moderate hypoxia 71–85% SpO_2_: 3 (1.3%)
	Hypert. Emergency >180 mmHg: 15 (7.8%)	Critical tachycardia > (220-age): 2 (1%)	Critical hypoxia <71% SpO_2_: 2 (1.1%)
Mean	140.75 mmHg	101.5 bpm	95.8% SpO_2_
SD	27.9 mmHg	20.3 bpm	5.2% SpO_2_
Med,	140 mmHg	100 bpm	97% SpO_2_
Range	80–250 mmHg	54–160 bpm	50–100 SpO_2_

### Laboratory measurements

Two hundred nineteen of 223 (98.2%) admissions received laboratory testing. 75% of those were performed within 2.5 h after arrival at the hospital, 97.8% later within the next 24 h, 1.8% on the next one day and 0.5% two days after the admission. The mean time from admission to arrival of blood in the laboratory was 3 h and 43 min (± 7 h and 1 min; median 1 h 15 min; range 9 min - 63 h 42 min). 155 patients (69.5%) had a second laboratory test after a mean of 35 h and 44 min (± 37 h 3 min; median 22 h 39 min, range 23 min - 227 h). 76 patients (34.1%) had a third laboratory test done after a mean of 77 h 12 min (± 57 h 56 min; median 55 h 16 min; range 8–328 h). The main laboratory parameters for each time point are provided in supplementary Table 1. Because of the heterogeneity of the timing of blood collection and the missing values, group comparison statistics such as repeated measure ANOVA or Friedman’s test were not applied. Hence, the laboratory dynamics reported here present trends, but not accurate kinetics.

### Complications, abnormal laboratory values and injuries

For the purpose of the study, a complication was defined as an event that requires additional attention, time and effort by the treating physician and nursing staff. Overall, 152 complications were documented, with the vast majority being minor injuries such as lacerations and bruises. Since serious complications were rare and the further clinical course usually uneventful, only troponin elevations were analyzed in greater detail. An overview is provided in Table 3.

**Table 3:** Overview of documented complications in association with GTCS

Complication	N	%
Injuries	102/223	45.7
Troponin elevations	9/75	12
Aggressive, delirious or psychotic behavior	14 / 223	6.3
Respiratory problems	7/223	6.3
Prolonged impairment of consciousness	11 / 223	4.9
Acute kidney injury	5 /136	3.7
Rhabdomyolysis	4/207	1.9

### Elevations of cardiac troponin I and myocardial ischemia.

**Fig. 2:**
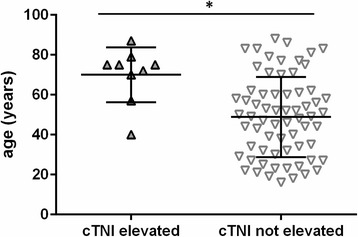
Patients with abnormally elevated cTNI levels were significantly older (*p* = 0.003) than patients with cTNI within normal levels. Wide bar: mean age. Error bars: standard deviation

During the time period covering this retrospective study, cTNI was no standard laboratory parameter for GTCS patients, but was performed on demand only. cTNI was measured once in 75 patients (34.2%), twice in 21 (9.6%) and thrice in 17 (7.7%) patients. The patients in whom cTNI was determined were on average 10 years older (41.7 ± 17. 7 years; 95% CI = 38.8–44.55 versus 51.4 ± 20.6 years; 95% CI = 46.7–56.16 years; *p* = 0.001) (Fig. 2), had more frequently preexisting heart disease (38.6% vs. 19.5%; *p* = 0.002) and suffered more often from prolonged impairment of consciousness/vigilance (9.3% vs. 2.7%; *p* = 0.046) and respiratory problems (6.7% vs. 1.4%; *p* = 0.044).

Mean initial cTNI was 0.06 ± 0.327 ng/ml (median: 0.02; range: 0.02–1.35 ng/ml). cTNI elevations above 0.05 n/ml were detected in 9 of 75 patients (12%). These patients received repeated cTNI measurements and a 12-channel-EKG and were presented to the local Department of Cardiology. A transthoracic echocardiography was performed in three and a coronary angiography in two cases in which EKG abnormalities were interpreted as most threatening. Only one patient complained of chest pain and shortness of breath. Seven of the nine patients had EKG changes (T-wave inversion in three cases, atrial fibrillation in three cases, one of which was new, delayed R-progression in one case). Five patients initially fulfilled NSTEMI-criteria with significantly rising cTNI values, while cTNI quickly fell in four other patients. In the two patients with coronary angiography, one was diagnosed with a non ST-elevation myocardial ischemia without coronary artery disease (CAD), while the other patient was diagnosed with an ST-elevation myocardial infarction and 3-vessels CAD. An overview of patients with troponin elevations is provided in Table 4.

**Table 4:** Overview of patients with cTNI elevations (ng/ml). 4 were diagnosed with NSTEMI, 1 with NSTEMI as a prodrome to a STEMI 4 days later. The NSTEMI diagnosis was “formal” and used the definition of rising cTNI levels in follow up laboratory tests. No patient except the one with a STEMI reported chest pain

No	Sex*	Age*	Max. cTNI	Epilepsy Hx	Medical Hx	EKG	Cardiac Dx	Other complications	outcome
1	1	70–79	0.68	EZ: temporal E: vascular	CNS-vasculitis, subdural hematoma, stroke, dementia, severe coronary artery disease with MI, stenting and aorto-coronary venous bypass, ICD, HTN, DM2, alcohol abuse	New AF	NSTEMI	No	Discharge home
2	1	80–89	0.14	EZ: unknown E: TBI	TBI, AF, HTN, bedridden	AF	AF, no ischemia	Delirium	Discharge home
3	2	70–79	0.38	EZ: unknown E: unknown	Hypothyroidism, Dyslipidemia	T-inversion	NSTEMI, received PTCA	Skull laceration	Discharge home
4	2	70–79	0.23	EZ: multifocal E: unknown	HTN, ICH, vascular dementia	Normal	No ischemia	No	Discharge home
5	1	70–79	0.34- > 15.7	EZ: unknown E: vascular	Stroke, PD, CHD with MI, polymyalgia, gastrointestinal bleeding	ST-depression, then elevation	NSTEMI as prodrome to STEMI 4 days later, received PTCA with DES	No	Transfer to cardiology, then home
6	2	50–59	3.85	EZ: unknown E: unknown	Craniofacial dysostosis	Delayed R-progression	NSTEMI	No	Discharge home
7	2	70–79	1.35	EZ: unknown E: TBI	TBI, stroke, coronary artery disease, NSTEMI	T-Inversion	NSTEMI	Sopor, intubation, skull laceration	Transfer to Geriatrics
8	2	70–79	0.12	EZ: unknown E: vascular	Stroke, AF, urinary tract infection	AF	AF, no ischemia	Sopor, intubation	Transfer to Geriatrics, death within a month
9	2	40–49	0.09	EZ: temporal E: unknown	Hypothyroidism	Normal	No ischemia	Facial bruising	Discharge home

AF = atrial fibrillation; DES = drug eluding stent; DM2, diabetes mellitus type 2; EZ = epileptogenic zone; E: etiology; HTN, arterial hypertension; Hx = history; ICH, intracranial hemorrhage; NSTEMI = non ST elevation myocardial infarction; PTCA = percutaneous, transluminal coronary angiography; STEMI = ST elevation myocardial infarction; TBI = traumatic brain injury. *Sex is not indicated and age range (instead of age) is given to avoid potential identification of the patients (according to BMC policy)

In summary, 12% of patients with cTNI measurements had elevations and 55.5% of them were diagnosed with a form of myocardial ischemia in the Department of Cardiology at the University Hospital Bonn. Patients with cTNI elevations were significantly older than those without (49 ± 20 years; 95% CI = 43.95–53.83 years versus 70 ± 14 years; 95% CI = 59.41–80.59; *p* = 0.005). Somewhat surprisingly, preexisting cardiovascular risk factors were not found to be significantly associated with cTNI elevations (30.3% vs. 33.3%; *p* = 1), most likely because of the small group size and the selection bias mentioned above. There was a trend towards an increased prevalence of cardiovascular diseases (66.7 vs. 34.8%; *p* = 0.08) in the group with cTNI elevations. Of note, one 40-year old patient without cardiovascular risk factors or cardiac disease was among the patients with cTNI elevations. Due to the small number of cTNI elevations, a formal regression analysis was not feasible.

### CK elevations and rhabdomyolysis

Determination of CK is part of our standard laboratory after seizures. Nevertheless, it was measured in only 207 out of 219 (94.5%) cases on the first day. 107 patients (48.9%) had one follow-up and 48 patients (21.9%) had two follow-up CK measurements. In 85 patients CK values (41.1%) were within the normal range. Mild CK elevations were found in 97 patients (<5× upper limit of normal, 46.9%), moderate elevations in 12 patients (5-10× upper limit of normal, 5.8%) and marked elevations in 9 patients (10-50× upper limit of normal, 4.3%). Severe rhabdomyolysis was observed in 4 patients (>50× upper limit of normal, 1.9%).

**Fig. 3:**
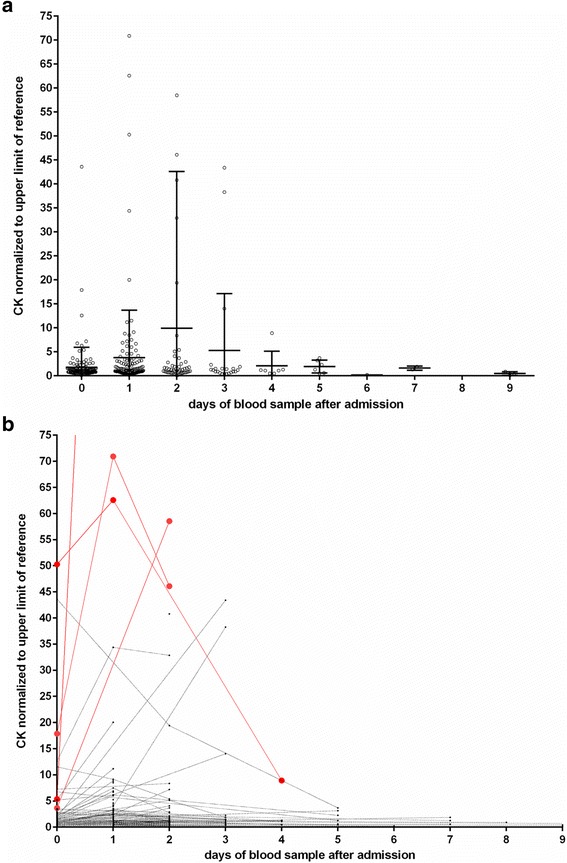
Time course of CK measurements for individual patients (**a**). An outlier with a CK 350 times above the upper limit of normal was clipped from the figure. The patients with severe rhabdomyolysis are labeled in red. Time course of CK measurements in the week after the emergency admissions (**b**). Wide bar: mean. Error bars: standard deviation. The actual measurements are normalized to sex- specific upper limits of the reference range

CK elevations occurred more frequently in males than in females (65.3% in males, 51.2% in females, *p* = 0.041) and tended to be more frequently found in patients with injuries (66.7%) than in those without (33.3%, *p* = 0.055). A trend of the CK time course is shown in Fig. 3a and b.

### Creatinine and acute kidney injuries

Serial measurements of creatinine (which allows calculation of AKIN-scores) were available in 136 patients. Three patients (2.2%) developed mild and 2 patients (1.5%) developed moderate acute renal injury. None of them went into frank acute renal failure. Only one of the 5 patients with AKI also had rhabdomyolysis, which indicated other mechanisms to be at play.

### Prolonged impairment of consciousness and postictal psychiatric problems

GTCS are commonly followed by a phase of impaired vigilance and consciousness with reorientation taking minutes to hours. In our patient group, 126 (56.5%) were fully reoriented when arriving at the ER. 13 patients (5.8%) were still obtunded, while the remaining 84 patients (37.6%) showed mild postictal confusion or disorientation that normalized to their usual baseline within some hours. Nine patients (4%) displayed postictal aggression, 4 (1.8%) were delirious and 1 (0.4%) psychotic.

### Respiratory complications

Seven patients (3.1%) developed respiratory problems (i.e. affection of pulmonary function). 1 patient (0.4%) developed dyspnea that was managed with noninvasive O_2_ supplementation, 2 patients developed (0.9%) aspiration pneumonia, and 4 (1.7%) required intubation.

### Injuries and radiologic exams

One hundred and two (45.7%) patients experienced additional injuries (Table 5). Injuries of the face, mouth, nose, tongue and skull were the most common complaints with bruises or lacerations of the head and face in 57 cases (25.5%), tongue/cheek-bites in 42 cases (18.8%), concussions in 3 cases (1.3%), epistaxis in 1 (0.4%) and tooth injuries in 1 case (0.4%). More severe injuries were 4 long bone or vertebral fractures (1.3%), 3 skull fractures (1.3%) and one severe traumatic brain injury with a traumatic, intracerebral hemorrhage (0.4%). One hundred and three patients underwent a cranial CT scan (46.2%), 7 underwent both conventional radiographies of their limbs and a cranial CT (33.1%), 1 patient only a conventional radiography (0.4%) and 4 a MRI (1.8%) (Table 6). Presumed head trauma with our without bruising and lacerations were the most common reason to conduct CT scans (*n* = 75, 58.1%), followed by real or presumed first seizures (*n* = 15; 11.6%), prolonged impairment of consciousness (n = 15; 11.6%) and Todd’s paralysis (*n* = 10; 7.8%). Overall, the 115 radiologic examinations ordered yielded pathological results in 8 cases (6.95%).

**Table 5:** Overview of documented injuries. TBI, traumatic brain injury. ICH, intracranial hemorrhage

No injuries	121 (54.3%)
Mouth/nose:	Tongue/cheek-bite: 42 (18.8%)
	Epistaxis: 1 (0.4%)
	Tooth injuries: 1 (0.4%)
head/face:	Bruise/laceration: 57(25.5%)
	Mild TBI: 3 (1.3%)
	Skull fracture: 3 (1.3%)
	TBI with ICH: 1 (0.4%)
Torso/limbs:	Bruise/laceration: 10 (4.5%)
	Fractures: 4 (1.8%)
radiographies:	129 (57.8%)
	Pathologic: 7 (6%)

**Table 6:** Overview of emergency radiographic tests applied

Imaging mode	N	%
Radiography alone	1 / 223	0.4
CT	103 / 223	46.2
Radiography and CT	7 / 223	3.1
MRI	4 / 223	1.8
Indications:	N	%
Head trauma	75 / 129	58.1
Prolonged alteration of consciousness	12 / 129	9.3
First seizure	15 / 129	11.6
Todd-phenomenon	10 / 129	7.8
Focal deficit	1 / 129	0.8
Known vasc, malformation with headache	3 / 129	2.3
Risk factors for hemorrhage	3 / 129	2.3
Reemergent seizure after long remission	3 /129	2.3
Aspiration	1 /129	0.8
history of brain abscess	1 /129	0.8
Backpain	1 /129	0.8
history of stroke	1 /129	0.8
unknown etiology	3 / 129	2.3

## Discussion

In our study population comprising 223 patients, 32 complications (14.3%, respiratory, mental state) and 114 injuries (51.1%) were diagnosed based on clinical examination, whereas 18 complications (8.2% of 219) were identified using laboratory values and 8 injuries were diagnosed with 115 imaging studies (6.95%). Hence, clinical exam revealed an important finding in 65.5% and ancillary tests in ~15%.

### cTNI

Elevations of cTNI may hint to the cardiac stress that epilepsy patients are exposed to during GTCS. Twelve percent of our patients displayed cTNI elevations, which is in line with most previous ER studies [17–20] reporting cTNI elevations in 6.5–28.6% of their patients. However, Woodruf [21], Alehan [22] Eskandarian [23] and Hajsadeghi [24] found no cTNI elevations in their patient groups, but these studies excluded patients with preexisting cardiovascular conditions or examined children and adolescents only. In contrast, our study had a bias towards testing sicker patients, since cTNI was ordered in elderly and multimorbid patients more often than in the young. Except for the video-EEG-monitoring study by Woodruff, most studies recruited their patients from large, interdisciplinary emergency rooms, while we only included patients who were finally admitted to our tertiary epilepsy center. While our patients had a history of epilepsy in 89.4% and new onset seizures in 10.6% only, first seizure patients constituted 42.8% - 68% of the patients in previous studies. Furthermore, despite careful efforts to identify and exclude patients with other conditions than epilepsy, retrospective ER studies may include a small number of false positives patients with psychogenic non-epileptic seizures and convulsive syncope in the putative seizure group. In contrast to other studies, our patient group did not include cases with acute symptomatic seizures due to vascular accidents, trauma, infection or other reasons and very few with alcohol-withdrawal related seizures, which may confound laboratory analyses. Thus, our study has a smaller sample size, but a higher proportion of preexisting epilepsy and very few acute symptomatic seizures, which may explain some of the differences to the previous studies. Some major findings of previous studies were, however, confirmed in our patient group. Age appears to be the most important and consistent risk factor to develop cTNI elevations after a seizure. To our surprise, we did not confirm cardiovascular risk factors and preexisting heart disease to be statistically significant risk factors, which may be attributable to the small sample size and selection bias. Most studies did not show evidence of cardiac ischemia in seizure-related cTNI elevations. Schneider and colleagues attributed a significant portion to Takotsubo cardiomyopathy [19], which we did not find, probably partially due to the infrequent transthoracic echocardiographies performed in our study. The diagnosis of NSTEMI was based on the laboratory criterion of rising cTNI as well as EKG criteria and clinical course. One patient had an NSTEMI at first and converted to a STEMI within 4 days, when PTCA confirmed CAD.

Chatzikonstantinou and colleagues assumed a temporal seizure onset to be one of the main risk factors for ictal cTNI release, based on imaging or interictal EEG- but not on video-EEG-findings [18]. The number of patients with a confirmed epileptogenic zone in our sample was too small to retest this hypothesis. However, the temporal lobe is the most common site of symptomatic seizures, which is the predominant epilepsy type in the elderly. One likely mechanism of seizure related cTNI release is relative ischemia, especially in preexisting CAD or other heart diseases, since a GTCS leads to simultaneous increase in pulmonary and systemic resistance, hypoxemia and catecholamine release [25], which can be compared to the situation in extreme athletic performance. In our cases with a quick return to a normal cTNI, a mechanism similar to cTNI release in vigorous exercise is assumed but cannot be proven at this point. After all, such cTNI elevations are more common in endurance sports [26] and less common in sprinters and other athletes with short but extreme demands to cardiac output, a situation more akin to a GTCS. These patients were discharged with a recommendation for a thorough cardiac workup but lost to follow up.

### CK

We found CK elevations in about two thirds of all GTCS-admissions, which is in line with data of previous studies [27–38]. Contrary to our assumptions, neither injuries nor multiple seizures were risk factors for increased CK values, but there was a clear trend towards higher CK values in injured patients. Chesson and Glotzner [28, 30] showed in earlier studies that injuries are no prerequisite to CK elevations. They and others hypothesized CK to rise especially after longer seizures and seizure series, for which we did not test, since the seizure duration was not recorded reliably. Thus, our study did not further illuminate the mystery why CK rises in some but not all GTCS seizures.

Severe rhabdomyolysis affected only 1.9% of our patients. This small number did not allow further analysis and assumptions on risk factors which could point to rhabdomyolysis on admission. Nevertheless, a high initial CK value appears to point towards an increased risk of severe rhabdomyolysis. Repetitive seizures, injuries or status epilepticus are no prerequisite for severe rhabdomyolysis, even though they are likely to elevate the risk. It would be interesting to see whether sensitive and specific cut-off values on initial CK could be identified as a warning sign in the development of rhabdomyolysis. Large, prospective studies would be needed to do so.

### Creatinine

While we did not encounter cases of acute renal failure in our sample, 3.6% developed mild to moderate acute renal injuries. Rhabdomyolysis is thought to be the most important risk factor for renal injuries after seizures [39–41]. Interestingly, in our patient sample, the mechanism was not related to rhabdomyolysis except in one patient. Hyperuricemia in the setting of postictal lactic acidosis is the likely, alternative mechanism, as others have shown on multiple occasions [42–44]. Since uric acid is not part of our standard laboratory measurements after seizures, no data are available to support this hypothesis. Few studies investigated postictal renal functions, since postictal acute renal failure is an overall rare event. Subtle renal dysfunction, however, was found in 8% of children with febrile convulsion in a study similar to ours [45].

### Prolonged impairment of consciousness and respiratory problems

Prolonged impairment of consciousness is readily detectable clinically and does not require laboratory testing. It is one of the risk factors for sudden unexpected death in epilepsy (SUDEP), presumably by reducing arousal function, protective brain stem reflexes and respiratory drive, thereby facilitating secondary asystole [25, 46]. Furthermore, a minor proportion of patients displayed respiratory problems that required specific treatment.

### Psychiatric problems

Aggressive, delirious, or psychotic behavior is recognizable clinically and does not necessarily require lab tests to detect. Postictal delirium can be difficult to differentiate from nonconvulsive status epilepticus, again not a laboratory diagnosis.

### Injuries and radiographies

Superficial injuries such as bruises and lacerations are very common in seizure patients, often requiring cooling or stitching of wounds. Only 6.95% of all radiographs revealed a relevant pathological finding, which is slightly worse then what was previously reported in the literature, where scans that will change acute management were found in 9–17% of adult and 3–8% of pediatric patients including first seizures and epilepsy [47]. If we looked only at head CT scans, the yield in our cohort with mostly known epilepsies was even lower, revealing 3 cases of skull fractures and one intracranial hemorrhage after 103 CCTs (3.9%), while 50% of 8 conventional radiographies showed a long bone or vertebral fracture.

### Study limitations

Due to its retrospective design our study has a number of limitations. First, the diagnosis of GTCS was based on observation and report of patients, relatives or other lay-witnesses only, which is an inherent source of error in all studies investigating ER admissions. The patients, however, were finally admitted to the Department of Epileptology where they underwent a more detailed history taking and subsequent epilepsy-related investigations (if epilepsy was not known), improving the level of certainty of the correct diagnosis. Second, by including only patients who were transported to the ER with emergency services, we aimed for higher rate of detected complications. Since patients who went home after the emergency room visit and patients who came without rescue services were not included, there is a supposed bias towards higher rates of complications. In general, ER studies may tend to investigate more severe cases than e.g. studies performed in a video-EEG telemetry unit. This potential bias may even strengthen the value of the study, as it magnifies the problem of rare postictal complications. Furthermore, both the timing and selection of laboratory measurements within our group were quite heterogeneous. The reasonable number of included patients and the real-life condition, however, allows the identification of clinically relevant risk factors in the ER setting. An echocardiography would have been desirable in all patients with cTNI elevations and has become our routine practice since. Finally, follow-up data on patients with signs of cardiac ischemia would be valuable but are lacking in our study.

## Conclusions

The actual yield of emergency laboratory and radiologic testing to detect severe complications of GTCS in adults with known epilepsy appears to be low, as cardiac, renal or muscular complications as well as severe injuries were found in only up to 2% of our patients. However, about 10% of the epilepsy patients who are referred to an ER following a GTCS suffered from at least one complication that requires further diagnostic and/or therapeutic interventions. Importantly, troponin elevations are one of the more common complications after emergency admissions due to GTCS, affecting about 10% of elderly patients. The most important risk factor is the age of the patient, suggesting that further cardiologic investigations are required in a subgroup of patients.

## Abbreviations

CAD: Coronary artery disease
CK: Creatine kinase
cTNI: Cardiac troponin I
ER: Emergency room
GTCS: Generalized tonic-clonic seizures
SUDEP: Sudden unexpected death in epilepsy
